# Microstructure and Arc Erosion Performance of CuCr50Ni_X_ Coatings by Infrared-Blue Hybrid Laser Cladding

**DOI:** 10.3390/ma19071389

**Published:** 2026-03-31

**Authors:** Yusen Duan, Xiuhua Guo, Jiang Feng, Chaomin Zhang, Kexing Song, Zhihua Wang, Kai Li, Yanyan Fan

**Affiliations:** 1 Henan Key Laboratory of Non-Ferrous Materials Science and Processing Technology, School of Materials Science and Engineering, Henan University of Science and Technology, Luoyang 471023, China; 2 Henan Key Laboratory of Advanced Conductor Materials, Henan Academy of Sciences, Zhengzhou 450046, China; 3 Henan Pinggao Electric Co., Ltd., Pingdingshan 467001, China

**Keywords:** infrared-blue hybrid laser cladding, CuCr50Ni coating, Ni addition, arc erosion resistance

## Abstract

**Highlights:**

- CuCr50Ni_X_ (X = 0, 0.1, 0.5, and 0.9 wt.%) coatings were fabricated on Cu via infrared-blue hybrid laser cladding.
- Trace Ni (0.5 wt.%) refines Cr phase and induces nanoscale Cr_7_Ni_3_ precipitates at Cr/Cu interfaces.
- Optimal coating achieves 174.2 HV_0.5_ hardness (149% higher than substrate) and 29.8% IACS conductivity.
- Arc erosion pits transform from deep localized (~37.7 μm) to shallow uniform (~9.6 μm) morphology.
- Ni addition strategy effectively balances electrical conductivity and arc erosion resistance.
- Cr_7_Ni_3_ nanoprecipitates enhance phase boundary stability and guide dynamic arc root movement.
- This study provides a novel surface alloying approach for high-performance electrical contact materials.
- The study demonstrates the potential of laser additive manufacturing for long-life vacuum interrupter components.

**Abstract:**

In this study, CuCr50Ni_X_ (X = 0, 0.1, 0.5, and 0.9 wt.%) coatings were successfully fabricated on a pure copper substrate via infrared-blue hybrid laser cladding. The effects of Ni addition on the microstructure, mechanical and electrical properties, and arc erosion resistance under 24 V/30 A conditions were systematically investigated. The results demonstrate that Ni refines the Cr phase and promotes the formation of a (Cr, Ni) solid solution and nanoscale Cr_7_Ni_3_ precipitates during non-equilibrium solidification. The coating with 0.5 wt.% Ni exhibits optimal comprehensive performance. It achieves a high microhardness of 174.2 HV_0.5_, representing a 149% increase compared to the copper substrate (72 HV_0.5_). Simultaneously, it maintains a good electrical conductivity of 29.8% IACS. Arc erosion morphology transforms from localized deep pits (CuCr50) to uniform, shallow characteristics (CuCr50Ni_0.5_), accompanied by reduced cathode mass loss. This enhanced performance is attributed to the refined and dispersed Cr distribution, which facilitates dynamic arc root movement, together with improved phase boundary stability conferred by the (Cr, Ni) solid solution and Cr_7_Ni_3_ precipitates. This work provides a novel strategy for developing high-performance, long-life electrical contact components through surface alloying design and laser additive manufacturing.

## 1. Introduction

Contact materials are the primary factor influencing the breaking performance and operational dependability of vacuum circuit breakers. Copper–chromium (CuCr) alloys are widely used in medium- and low-voltage vacuum interrupters, owing to their combination of favorable properties, including high electrical and thermal conductivity, good mechanical strength, and high resistance to arc erosion and welding [1,2,3,4]. Power systems are evolving to higher voltage levels and confronting rigorous demands related to energy conservation, environmental protection, equipment miniaturization, and elevated current density. Consequently, conventional CuCr contact materials face considerable challenges when subjected to prolonged high-current operation and repeated arcing. On the one hand, high-arc energy induces significant erosion, material transfer, and potential welding on contact surfaces, undermining contact stability and longevity [5,6,7]. Conversely, augmenting chromium content to improve voltage and arc resistance frequently diminishes electrical and thermal conductivity, restricting usability under elevated load conditions [8,9,10]. Consequently, preserving the elevated conductivity of CuCr alloys while enhancing their resistance to arc erosion and mitigating fusion welding tendencies has emerged as a pivotal focus in the advancement of high-performance electrical contact materials.

Various studies have revealed that, as an advanced method of surface modification, laser cladding provides numerous benefits over conventional surface treatments such as cold spraying, vapor deposition, and electroplating [11,12,13,14,15]. This method can significantly increase the service life of copper alloys while largely preserving their high electrical conductivity owing to its excellent coating qualities, high material utilization, and strong bond strength [16]. In particular, infrared-blue hybrid laser cladding technology has emerged in recent years; this technology uniquely combines the grain refinement effect of rapid solidification [17,18,19] with the process stability conferred by dual-wavelength energy coupling [20,21]. The blue laser substantially increases the absorptivity of the copper substrate, circumventing the long-standing challenge of processing copper with conventional infrared lasers [22,23,24]. Concurrently, the infrared laser supplies sufficient thermal input to establish a high-strength metallurgical bond between coatings and substrate. This offers a dependable approach for producing high-performance CuCr alloy coatings on pure copper or copper alloy surfaces, potentially addressing the technical challenge of balancing conductivity and durability in copper-based electrical contact materials.

Infrared-blue hybrid laser cladding technology has made considerable advancements in enhancing the surfaces of copper alloys. Tan et al. [25] effectively generated high-quality CoCrFeNi coatings on copper alloys using this approach, noting that the hybrid laser markedly improved molten pool stability and the quality of coating formation, leading to dependable coating performance. Wang et al. [26] utilized infrared-blue hybrid laser cladding to apply AlCoCrFeNiCu_x_ high-entropy alloy coatings onto copper alloy surfaces. By regulating variations in copper concentration, they examined the mechanism that dictates the tribological wear characteristics of the coated substrate, thereby improving both current-carrying friction performance and electrical conductivity. Liu et al. [27] utilized infrared-blue hybrid laser cladding to apply CoCrFeNiX (X = Mo, Ti, W) high-entropy alloy coatings on copper alloys, significantly improving coating hardness and wear resistance. The aforementioned research demonstrates that infrared-blue hybrid laser cladding is applicable for the preparation of coatings on copper alloy surfaces. Current research on infrared-blue hybrid laser cladding technology for copper alloys predominantly emphasizes coating preparation to improve surface wear resistance, with less documentation on the development of ablation-resistant coatings for copper alloy surfaces.

Research indicates [28,29,30] that the introduction of Ni significantly enhances the mutual solubility between Cu and Cr phases while stabilizing the Cr phase structure through solid solution strengthening. This mechanism beneficially contributes to improved dielectric strength and arc erosion resistance. For instance, Wei et al. [31] synthesized CuCr45 and CuCr42.7Ni2.3 alloys using mechanical powder mixing and vacuum hot-press sintering. The inclusion of Ni markedly inhibited liquid phase separation at high temperatures while facilitating Cr phase refining. Zhang et al. [32] successfully synthesized CuCr25Ni0.5 alloy via vacuum induction melting, reporting that Ni addition not only induced pronounced refinement and spheroidization of the Cr phase but also substantially enhanced the breakdown voltage strength. Li et al. [33] employed medium-frequency induction melting under ambient atmosphere to prepare Cu-1.16Ni-0.36Cr alloy. Following tensile deformation at 700 °C, they observed the precipitation of metastable Cr_7_Ni_3_ phases with an average size of approximately 10 nm, which effectively pinned dislocations and suppressed grain boundary migration, thereby markedly enhancing high-temperature mechanical performance. Previous studies have demonstrated that while Ni significantly refines Cr, its concentration exceeding 0.5 wt.% in CuCr alloys induces a sharp decline in electrical conductivity. Consequently, three distinct Ni concentrations (0.1, 0.5, and 0.9 wt.%) were selected to systematically investigate its effects, from trace to excessive addition during laser cladding, on microstructural evolution, property modification, and ablation resistance, while minimizing the trade-off with electrical conductivity.

In this study, CuCr50Ni_X_ (X = 0, 0.1, 0.5, and 0.9 wt.%) coatings were prepared on a pure copper substrate using infrared-blue hybrid laser cladding. The coaxial hybrid laser configuration was employed to overcome the challenges associated with laser processing of copper alloys. This approach aims to address the issues faced by conventional CuCr alloys under high-voltage and high-current conditions, such as Cr phase coarsening and insufficient arc erosion resistance. The microstructure and fundamental properties of the CuCr50 composite coating were compared in relation to the addition of Ni elements. The study concentrated on the functions of the (Cr, Ni) solid solution and Cr_7_Ni_3_ precipitation phase under the rapid solidification conditions of laser cladding. The ablation behavior and failure mechanisms of the coatings under different current settings were meticulously examined. The research demonstrated the essential regulatory function of nanoscale Cr_7_Ni_3_ precipitates at Cr phase boundaries in governing arc behavior. These findings are expected to inform the development of high-performance reinforced coatings for contact materials in copper alloy vacuum interrupters.

## 2. Materials and Methods

### 2.1. Raw Materials

**Figure 1:**
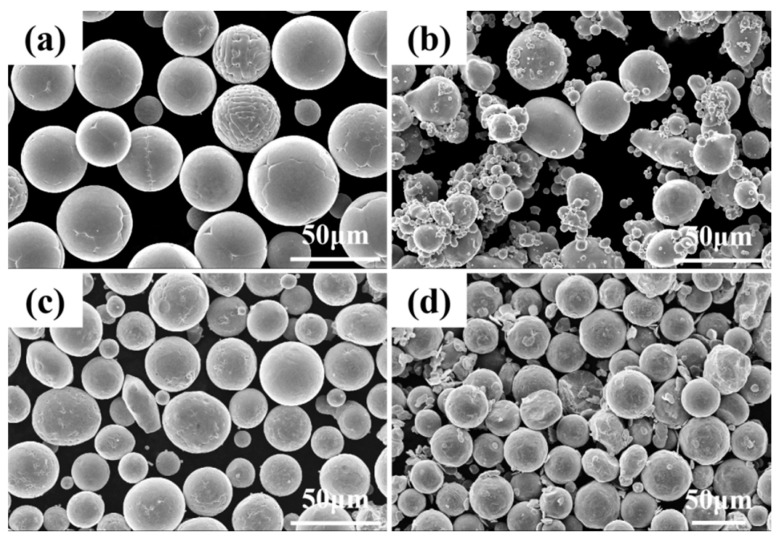
SEM images showing the raw material characteristics: (**a**) spherical Cu powder; (**b**) spherical Cr powder; (**c**) spherical Ni powder; and (**d**) CuCr50Ni mixed powder.

Figure 1 displays SEM images of the powders utilized in this experiment. The raw powders were Cu powder, Cr powder, and Ni powder (purity 99 wt.%, particle size 13–50 μm, Guangdong Stardust Technology Co., Ltd., Foshan, China). A commercially available pure copper plate with a size of 50 × 50 × 10 mm was selected as the substrate. The CuCr50Ni powders were prepared by adding 0.1, 0.5, and 0.9 wt.% Ni to the base CuCr50 composition, with the Cu:Cr mass ratio kept constant at 1:1. These mixtures were milled in a GMS5-4 jar mill at 80 r/min for 8 h, with a ball-to-powder mass ratio of 5:1 (agate balls to mixed powder). As shown in Figure 1d, the majority of particles maintained a spherical morphology and demonstrated good flowability. Before the experiment, the powders were dehydrated in a vacuum oven at 80 °C for 1 h to eliminate moisture.

### 2.2. Preparation of Laser Cladding Coatings

**Figure 2:**
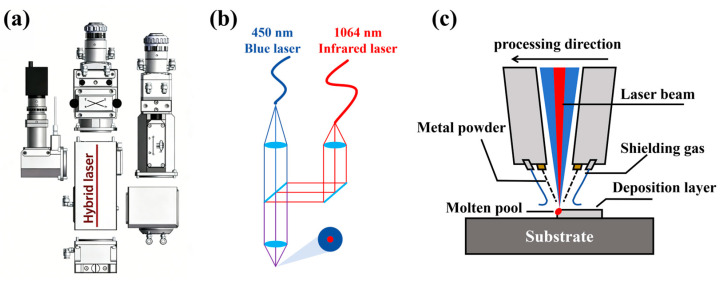
Schematic diagram of infrared-blue hybrid laser cladding: (**a**) laser head module diagram; (**b**) hybrid laser optical path diagram; (**c**) coating preparation schematic diagram.

As illustrated in Figure 2, the laser cladding experiments were carried out using a six-axis robot (FANUC, Oshino, Japan) in conjunction with a 2000 W blue laser (445 nm, Laserline, Mülheim-Kärlich, Germany) and a 6000 W fiber infrared laser (1064 nm, Keplin Photoelectric Co., Ltd., Beijing, China). The infrared laser had a spot size of 0.3 mm, whereas the blue laser had a spot size of 2 mm. The axes of both laser beams spatially coincided; the laser module used (Luoyang Aobote Intelligent Equipment Co., Ltd., Luoyang, China) and the optical path diagram are shown in Figure 1a,b. To protect equipment and mitigate laser reflection resulting from copper’s high reflectivity, the infrared laser power was restricted to 2500 W, while the blue laser power was limited to 2000 W due to equipment limitations. Meanwhile, during the formal experiment, the cladding head should be tilted 5° relative to the substrate for the test to avoid the feedback of reflected laser caused by vertical incident. Argon served as both the powder delivery gas and the shielding gas, flowing at 12 L/min and 10 L/min, respectively, ensuring that the shielding gas did not displace the powder. The mixed powder was delivered from a lateral nozzle. Before the experiment, the laser beams were concentrated at the center of the powder stream and the six-axis robot was controlled to move the laser to adjust its position to 8 mm above the base. The substrate surface was ground with a series of sandpapers from W28 to W5 to remove surface oxides and obtain a uniformly rough surface (Ra ≈ 1.2–1.8 μm), thereby improving the laser energy absorption efficiency [34]. The substrate remained immobile throughout the cladding procedure. Notably, the scanning strategy along the cladding direction was Zigzag. Based on the previous single-pass cladding experiments and research in the literature [35,36,37], other experimental parameters are shown in Table 1.

### 2.3. Experimental Characterization

The microstructure of the coating was examined in this experiment using a tungsten filament scanning electron microscope (SEM, JSM-IT100, JEOL, Tokyo, Japan). Furthermore, before SEM analysis, the polished cross-sectional samples were etched with FeCl_3_ reagent for 5–10 s. The phase composition of the cladding layer was analyzed by X-ray diffraction (XRD, D8-Advance, Bruker, Billerica, MA, USA) over a 2θ range of 30–100° with a step size of 0.02°. The elemental distribution inside the samples was examined by energy-dispersive X-ray spectroscopy (EDS). Transmission electron microscopy (TEM, JEM-2100, JEOL, Tokyo, Japan) was utilized to examine nanoscale precipitates, complemented by selected-area electron diffraction analysis (SAED). TEM specimens were then prepared by focused ion beam (FIB) from the middle region of the cladding layer at a consistent depth. The cladding samples were cut into rectangular specimens of 10 × 15 × 10 mm using a wire cutting machine. The Vickers hardness was assessed on the polished cross-section of the cladding layer using an HVS-1000A digital Vickers hardness tester (HST Group, Jinan, China) with a load of 500 g for a duration of 10 s. Hardness measurements were performed along the depth direction of the coating cross-section at intervals of 0.1 mm. At each indent, three repeated measurements were taken, and the average value was used as the final hardness to minimize measurement errors. The cladding surface was ground and polished prior to conductivity measurements being taken. The measurement of electrical conductivity was conducted utilizing an eddy current conductivity meter (model Sigma2008B1, Xiamen Tianyan Instrument Co., Ltd., Xiamen, China). To ensure accuracy, each conductivity measurement was repeated five times, and the average value was reported.

### 2.4. Electrical Contact Testing

**Figure 3:**
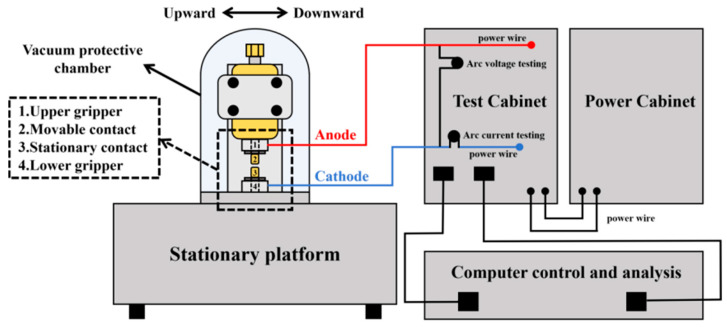
Schematic of the JF04D electric testing system.

A JF04D electrical contact tester (Kunming Precious Metals Research Institute, Kunming, China) equipped with a vacuum chamber was used to simulate the operating environment of a vacuum circuit breaker, as illustrated in Figure 3. The cladding specimens were machined into cylindrical anodes and cathodes with a diameter of 3.8 mm and a length of 10 mm. The surfaces of both the anode and cathode were mechanically polished to achieve a surface roughness of less than 0.25 μm. Each contact pair was subjected to 1000 cycles at a constant 24 V DC, with contact force ranging from 0.4 to 0.7 N by a precision force sensor. All test parameters were chosen in accordance with the instrument specifications. The tests were conducted at room temperature. The testing system automatically recorded data including arc energy and arc duration. Mass measurements before and after contact testing were performed using an electronic balance (FA2004B, Shanghai Yoke Instrument Co., Ltd., Shanghai, China). The surface morphology of ablated specimens was examined by SEM. Three-dimensional topography was characterized using a profilometer (Sensofar S neox, Sensofar Metrology, Barcelona, Spain) with a measurement area of 4 mm × 4 mm and a resolution of 1224 × 1024 pixels.

## 3. Results and Discussion

### 3.1. Microstructure and Physical Properties of the Coating

**Figure 4:**
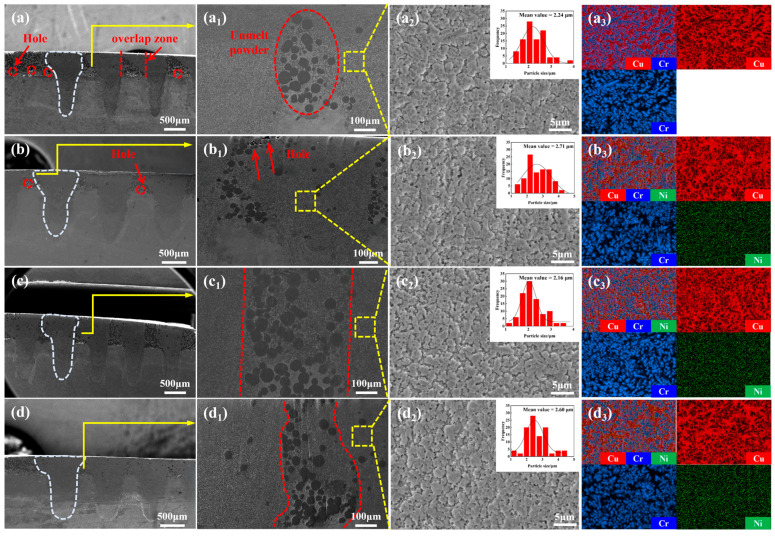
Cross-sectional morphology and microstructure analysis of CuCr50Ni_X_ (X = 0.0, 0.1, 0.5, 0.9 wt.% Ni) coatings: (**a**–**d**) overall cross-sectional morphologies, (**a_1_**–**d_1_**) magnified microstructures of the overlap zones, (**a_2_**–**d_2_**) local magnified microstructures and Cr phase particle size distribution, and (**a_3_**–**d_3_**) EDS elemental mapping of the corresponding regions.

Figure 4 presents the microstructure, grain size analysis diagram, and EDS mappings of the cross-section for the CuCr50Ni coating with varying Ni contents. As shown in Figure 4a–d, the coatings exhibit a tack-shaped bonding morphology with the pure Cu substrate. The bottom of the molten pool penetrates into the substrate, forming the “tack head”, while the spread cladding layer constitutes the “tack cap”. This morphology originates from the keyhole effect [38] induced by the high energy density at the center of the infrared Gaussian beam. Specifically, the high-energy-density infrared laser rapidly creates a depression on the molten pool surface, which further develops into a deep and narrow keyhole. The infrared laser beam undergoes multiple reflections within this keyhole, significantly enhancing the total absorption rate of laser energy by the molten pool. Studies have shown that under deep and narrow keyhole conditions, the maximum absorption rate can reach 93%, approaching complete absorption [39]. This keyhole effect typically indicates a strong metallurgical bond between the cladding layer and the substrate. Significantly, in contrast to samples without Ni addition (Figure 4a) and those with minimal Ni concentration (Figure 4b), a small number of pores are observable at the cladding–substrate interface. Nevertheless, an increase in Ni content markedly diminishes such defects (Figure 4c,d), suggesting that the addition of Ni enhances interface integrity. Figure 4(a_1_–d_1_) illustrate enlarged perspectives of the overlap zones associated with Figure 4a–d. The overlap zone, consisting of unmelted coarse Cr particles inside a Cu matrix, is present between the cladding tracks of samples with varying Ni concentrations, with no discernible second-phase precipitation detected. Figure 4(a_2_–d_2_) illustrate the microstructural study and the distribution of Cr phase grain sizes within the coating. The coating predominantly consists of dense cellular Cr phases, exhibiting no observable porosity or cracks. The comparative examination of the mean grain size of the Cr phase reveals that the addition of Ni markedly affects the size evolution of the Cr phase. As the Ni content rises, the Cr phase size exhibits an initial increase followed by a decrease. Significantly, with a Ni content of 0.5 wt.%, the mean Cr grain size is minimized at 2.16 ± 0.15 μm (Figure 4(c_2_)), representing a 3.5% decrease relative to the mean Cr grain size in the absence of Ni addition. This confirms that appropriate Ni addition effectively refines the Cr phase. The EDS mapping (Figure 4(a_3_–d_3_)) further reveals a well-defined interpenetrating network between Cr and Cu phases without notable macrosegregation, confirming that the dual-phase separation characteristic of the CuCr alloy is well preserved upon Ni doping [40]. The Cr phase forms as a continuous skeletal structure, whereas the Cu phase occupies the interstitial voids within the Cr framework. Meanwhile, the addition of Ni does not influence the distribution behavior of Cr. Ni is uniformly distributed without detectable Ni-enriched phases or segregation zones, confirming complete solid solution of Ni in the CuCr50 alloy. The microstructural features result mainly from the rapid cooling and solidification of the molten pool, which limits the long-range diffusion of solute atoms, thus inhibiting the nucleation and growth of intermetallic compounds and facilitating the formation of supersaturated solid solutions [41]. The lattice constant of Ni (0.352 nm) is close to that of Cu (0.361 nm), facilitating the formation of a continuous solid solution between Ni and Cu owing to their infinite mutual solubility. Notably, Ni may form Cr-Ni solid solutions with Cr [42], which alleviates lattice mismatch and thermal expansion disparities between Cu and Cr, mitigates internal stress following rapid laser solidification, and enhances coating integrity. This explains the near-complete absence of interfacial voids between the CuCr50Ni cladding and the substrate. In summary, Ni serves to inhibit the coarsening and segregation of the Cr phase during rapid solidification, thereby contributing to grain refinement.

**Figure 5:**
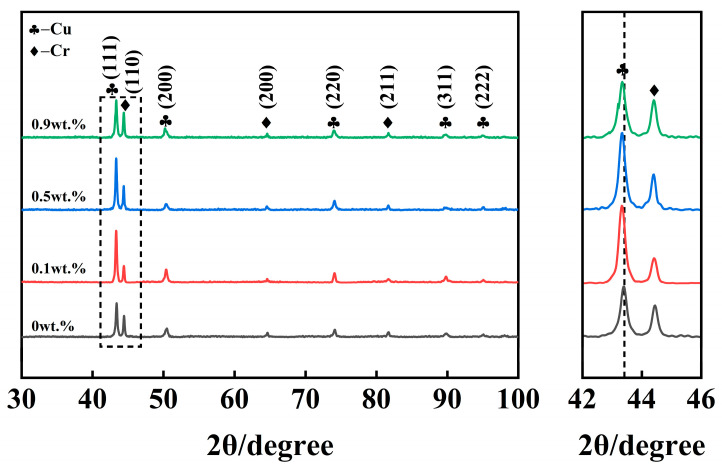
XRD patterns of CuCr50 coatings with different Ni contents.

Figure 5 shows the XRD patterns of CuCr50 coatings with different Ni contents. The positions of the principal Cu diffraction peaks shifted toward lower angles with increasing Ni content. This indicates that the content of atoms dissolved in the matrix varies. Ni elements dissolve into the Cu matrix, causing the Cu peak to shift to the left. To determine the effect of Ni dissolution into the copper matrix on lattice distortion, XRD diffraction peaks were analyzed using Jade 6. We used the Bragg equation (2dsinθ = nλ, where d represents the interplanar spacing, θ denotes the diffraction angle, n is the diffraction order, and λ is the radiation wavelength (λ = 0.154056 nm)). Calculations were conducted for the interplanar spacing d, lattice constant a, and relative distortion Da of the Cu-rich phase in CuCr50Ni. Applying the (111) Cu peak as an example, the findings are displayed in Table 2. As the Ni content increased from 0.0 to 0.9 wt.%, the lattice constant a increased progressively from 0.38836 nm to 0.38891 nm, with corresponding relative distortions Da of 0, 0.11072%, 0.12102%, and 0.14162%. The interplanar spacing d also increased accordingly. This indicates that Ni addition enhances lattice distortion in the coatings, which increases resistance to dislocation motion and induces solid solution strengthening, thereby improving mechanical properties [43]. The leftward shift in XRD diffraction peaks significantly increases with rising Ni content, directly reflecting the cumulative lattice strain effect induced by enhanced Ni solid solution solubility. This solid solution strengthening behavior indicates that an appropriate amount of Ni (0.5 wt.%) can achieve effective strengthening through lattice distortion, whereas excessive Ni may induce excessive lattice distortion and localized stress concentration, thereby weakening the strengthening and toughening effect.

**Figure 6:**
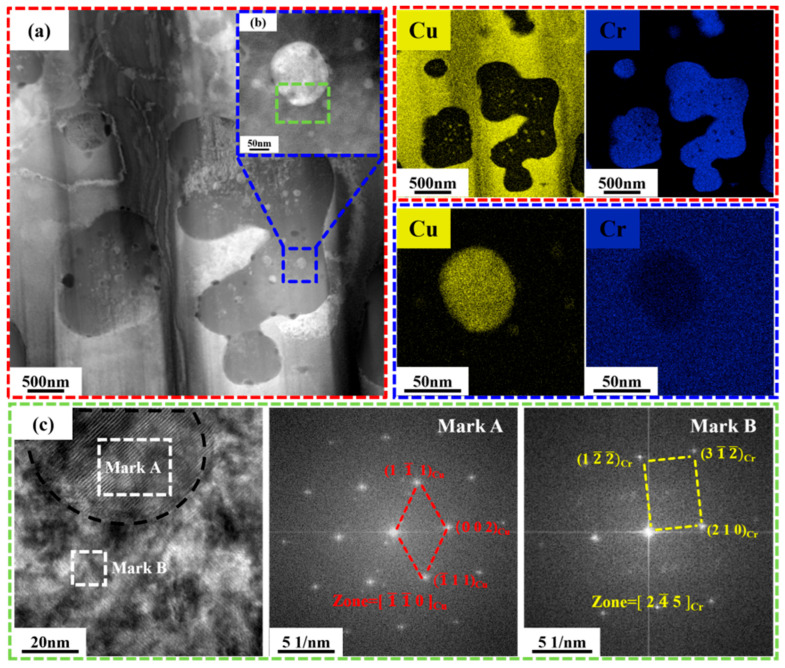
TEM analysis of CuCr50 clad layer: (**a**) HAADF and EDS images of the CuCr50 clad layer region; (**b**) HAADF and EDS images of the phase in the blue area of (**a**); (**c**) HRTEM of the green area in (**b**) and corresponding FFT images.

Transmission electron microscopy (TEM) was performed on the coatings to investigate their microstructure. Samples were taken from the middle region of the cladding layer to ensure consistency. To verify the uniformity of the microstructure, at least three different areas were examined for each specimen. Figure 6 presents transmission electron microscopy analyses of the CuCr50 coating (the middle regions), including high-angle annular dark-field (HAADF) and associated EDS images, high-resolution transmission electron microscopy (HRTEM) images, and corresponding selected-area electron diffraction (SAED) patterns. As shown in Figure 6a, the body-centered cubic (BCC) Cr phase (200–500 nm) is uniformly dispersed within a continuous Cu-rich matrix. Corresponding elemental mapping further confirms the spatial distribution of Cu and Cr phases. Notably, nanoscale precipitates (30–80 nm) are dispersed within the Cr phase. Dark-field imaging and elemental mapping of the region in Figure 6b confirm that these nanoparticles consist solely of Cu. The formation of this unique nanostructure is primarily attributed to the extremely high cooling rate (up to 10^6^ K/s) inherent in laser cladding. Under rapid solidification, the melt enters the immiscible region of the Cu-Cr system upon cooling below the liquidus temperature. Although the thermodynamic driving force favors extensive liquid phase separation and the formation of coarse Cr-rich and Cu-rich phases, the exceptionally high cooling rate substantially suppresses the nucleation, growth, and coarsening of the Cr phase, as well as the complete segregation of the two phases. During this highly non-equilibrium solidification process, Cr nucleates preferentially and grows rapidly, trapping residual liquid Cu that has not yet fully diffused. Additionally, the limited mutual solubility between Cu and Cr at elevated temperatures enables the retention of solute atoms upon rapid cooling, thereby further stabilizing the metastable two-phase microstructure. As a result, laser cladding produces an interwoven nanoscale architecture in which Cu and Cr phases mutually encapsulate one another. This configuration not only substantially increases the interfacial area but also enhances metallurgical bonding between phases, thereby establishing a microstructural foundation for improved material performance.

**Figure 7:**
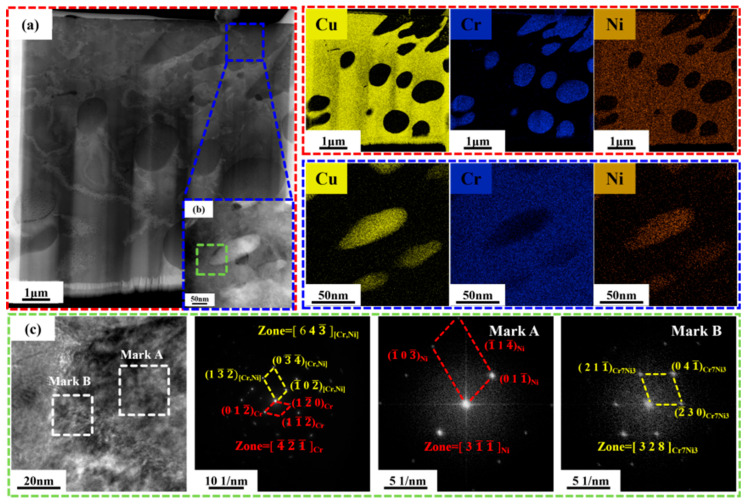
TEM analysis of CuCr50Ni_0.5_ clad layer: (**a**) HAADF and EDS images of the CuCr50Ni_0.5_ clad layer region; (**b**) HAADF and EDS images of the phase in the blue area of (**a**); (**c**) HRTEM of the green area in (**b**) and corresponding FFT images.

Figure 7 presents TEM analysis of the CuCr50Ni coating (the middle regions) with a Ni content of 0.5 wt.%, including HAADF images, corresponding EDS elemental maps, HRTEM images, and SAED patterns. As shown in Figure 7a, the microstructure features a continuous Cu matrix with uniformly embedded cellular Cr phases (200–500 nm), within which finely dispersed Cu nanoparticles (30–80 nm) are distinctly observed. Corresponding EDS mapping confirms the spatial distribution of Cu, Cr, and Ni, revealing that Ni is uniformly dispersed throughout the matrix without discernible segregation. Figure 7b presents a magnified dark-field image of the local region outlined in Figure 7a. Corresponding EDS analysis reveals that, in addition to the aforementioned Cu phase, a dispersed Ni phase with a size distribution concentrated in the range of 30–50 nm is also present within the Cr phase. Figure 7c displays an HRTEM image and corresponding FFT diffractogram of the selected region in Figure 7b. Indexing of the diffraction pattern confirms the coexistence of a (Cr, Ni) solid solution and an independent Ni phase within this microregion. This coexistence implies that Ni atoms are forced into the Cr lattice by the ultra-high cooling rate characteristic of laser cladding, creating a supersaturated solid solution and preventing the precipitation of coarse, discrete Ni phases. Notably, at the Cu-Ni/Cr interface region (indicated as Mark B in Figure 7c), fine plate-like precipitates measuring approximately 5–15 nm were observed. Diffraction analysis identified these precipitates as the Cr_7_Ni_3_ phase with a zone axis of [3,2,8]. Slight shifts in the diffraction spots corroborate the addition of Ni atoms into the Cr lattice, inducing discernible lattice distortion. The formation of this precipitate is also attributable to non-equilibrium solidification resulting from the extremely high cooling rates inherent in laser cladding. Typically, these intermetallics necessitate post-aging to induce precipitation. In contrast, the rapid solidification conditions utilized in this study induce local compositional variations and significant undercooling, which facilitate solute partitioning and ordering at the final phase of solidification, resulting in nanoscale Cr_7_Ni_3_ precipitates within the clad layer without subsequent heat treatment.

**Figure 8:**
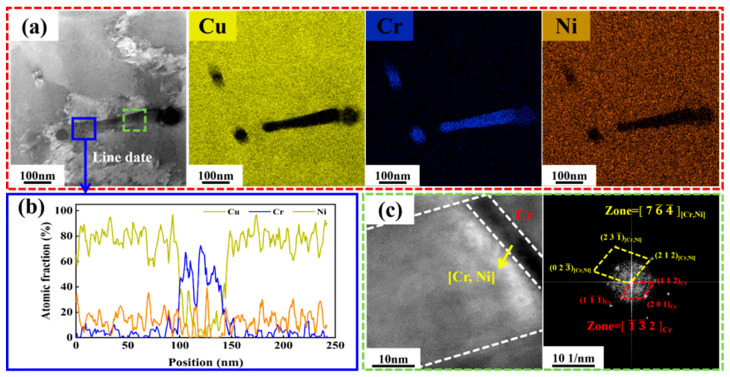
TEM analysis of another region of the CuCr50Ni_0.5_ clad layer: (**a**) HAADF and EDS images of the CuCr50Ni_0.5_ clad layer region; (**b**) line scan of the blue region in (**a**); (**c**) HRTEM of the green area in (**b**) and corresponding FFT images.

Figure 8 shows detailed microstructural characterization of another representative region within the CuCr50Ni coating (the middle regions) containing 0.5 wt.% Ni, including an HAADF image, corresponding EDS elemental maps, HRTEM images, and SAED patterns. As shown in Figure 8a, the Cr phases display a rod-like morphology, while Ni is dispersedly distributed at both the lateral and terminal regions of the rods. The line scan analysis results for this location (Figure 8b) further confirm that the rod-like phase consists primarily of Cr, whereas Ni displays a consistent distribution throughout the cross-section, suggesting that Ni atoms have dissolved into the Cr lattice. The HRTEM image of the corresponding region (Figure 8c), together with its SAED pattern indexed as a (Cr, Ni) solid solution, provides further evidence for the solid solution behavior of Ni within the Cr phase. Based on the foregoing observations, the characteristics of non-equilibrium solidification microstructures arising during rapid solidification of laser-clad CuCr50 and CuCr50Ni can be summarized as follows: The first is an encapsulated structure in which nanoscale Cu particles are embedded within the Cr phase (Figure 6). The second is the formation of a (Cr, Ni) solid solution resulting from the forced dissolution of Ni into the Cr lattice, and the consequent precipitation of Cr_7_Ni_3_ with a long-range ordered superlattice (Figure 7 and Figure 8). These characteristic microstructures constitute direct microscopic evidence that the extreme cooling rates inherent in laser cladding effectively suppress solute diffusion and retain the high-temperature mutual solubility state. This contrasts fundamentally with the equilibrium microstructures obtained via conventional powder metallurgy or induction melting routes followed by aging treatments.

**Figure 9:**
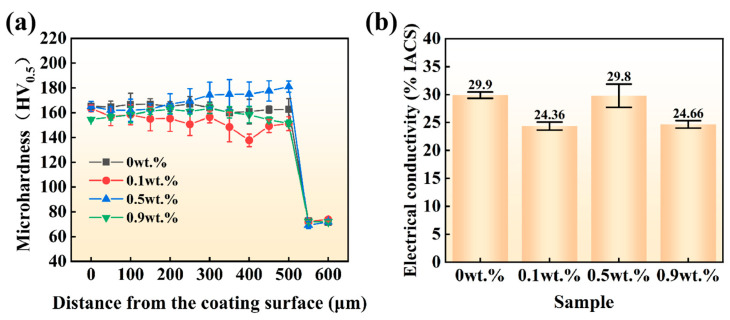
Cross-sections of CuCr50Ni coatings with different Ni contents: (**a**) Vickers hardness; (**b**) electrical conductivity.

Figure 9 presents the cross-sectional Vickers hardness and electrical conductivity of the coatings with different Ni contents. Both hardness and electrical conductivity exhibit a non-monotonic dependence on Ni content, characterized by an initial decline, followed by an increase and a subsequent decrease. Specifically, at a Ni content of 0.5 wt.%, the coating exhibits a maximum microhardness of 174.2 HV_0.5_, indicating an approximate 149% improvement compared to the Cu substrate (72 HV_0.5_), while maintaining a conductivity of 29.8% IACS (Figure 9a,b). In contrast, a minor Ni addition (0.1 wt.%) results in a general hardness degradation, with the curve dropping further in the near-substrate region, and offers no significant gain in conductivity. While excessive Ni (0.9 wt.%) likewise induces simultaneous reductions in both hardness and conductivity, the magnitude of decline is less pronounced than that observed with trace Ni addition. It is worth noting that the hardness distribution along the depth direction varies significantly with Ni content. For the 0.5 wt.% Ni sample, the hardness gradually increases with increasing distance from the surface and reaches a peak near the heat-affected zone. This is attributed to the higher cooling rate at the bottom of the molten pool, which leads to a more pronounced grain refinement strengthening effect. In contrast, the 0.1 wt.% Ni sample exhibits a decrease in hardness with increasing depth, due to insufficient solid solution strengthening and higher dilution at the pool bottom. For the 0.9 wt.% Ni sample, the hardness also decreases with depth, which can be explained by excessive lattice distortion, agglomeration of the Cr phase, and coarsening of grains at the bottom. These phenomena are closely associated with the solid solution behavior of Ni in the Cu-Cr system and the resultant microstructural evolution. The addition of an optimal quantity of Ni (0.5 wt.%) facilitates the development of (Cr, Ni) solid solutions and markedly enhances the microstructure by inhibiting Cr phase coarsening and segregation, resulting in a reduction in the average Cr particle size by around 4%. This is consistent with the grain refinement strengthening mechanism elucidated by the Hall–Petch relationship [44,45]. Meanwhile, the homogeneous solid solution of Ni in the Cu matrix induces moderate lattice distortion, contributing to solid solution strengthening. However, an insufficient Ni content (0.1 wt.%) fails to provide adequate solid solution strengthening; instead, it induces hardness degradation due to weak interfacial bonding and local stress concentration. Conversely, excessive Ni (0.9 wt.%) induces excessive lattice distortion, weakening the plasticity of the Cu matrix and thus degrading the hardness.

As a typical immiscible alloy, CuCr exhibits extremely limited solid solubility between its components. Under the assumption of complete immiscibility, the overall electrical conductivity of CuCr alloys can be defined by the average of the conductivities and volume fractions of the two pure metals [46]. The expression is as follows:

(1) $$\sigma_{CuCr}=\;\sigma_{Cu}\cdot A_{Cu}+\;\sigma_{Cr}\cdot A_{Cr}$$

(2) $$\sigma_{CuCr}=\sigma_{Cu}-\left(\sigma_{Cu}-\sigma_{Cr}\right)\cdot \omega_{Cr}\cdot \frac{\rho_{Cu}}{\left(\omega_{Cr}\cdot \rho_{Cu}\right)}+\left(1-\omega_{Cr}\right)\cdot \rho_{Cr}$$

where σ_CuCr_ denotes the overall conductivity, σ_Cu_ represents the conductivity of pure copper, σ_Cr_ denotes the conductivity of pure chromium, A_Cu_ is defined as the volume percentage of copper in the alloy, A_Cr_ is the volume fraction of chromium, ω_Cr_ indicates the mass fraction of chromium, ρ_Cu_ refers to the density of copper, and ρ_Cr_ refers to the density of pure chromium.

According to alloy conduction theory, the electrical conductivity of alloys is primarily influenced by lattice distortion within the solid solution of CuCr alloys, as well as defects such as grain boundaries, vacancies, impurity phases, and pores. When trace or excessive amounts of Ni are added, its solid solution in the Cu matrix introduces additional lattice distortion, increasing the probability of electron scattering. This reduces σ_Cu_ and ultimately degrades the electrical conductivity. At a Ni content of 0.5 wt.%, the refining effect of Ni reduces the size and improves the distribution uniformity of Cr phase particles. This reduces the electron scattering effect at phase boundaries, allowing the conductivity to recover to a level close to that without Ni addition. With an increase in Ni concentration, several Ni atoms uniformly dissolve into the Cu lattice, significantly increasing lattice distortion. This enhances electron scattering during motion and consequently reduces electrical conductivity. However, owing to the homogeneity of this distortion, the severe scattering typically caused by localized, highly distorted regions (characteristic of trace Ni additions) is absent. Thus, the conductivity drop in the CuCr50Ni_0.9_ coating is significantly less pronounced than that in CuCr50Ni_0.1_.

**Figure 10:**
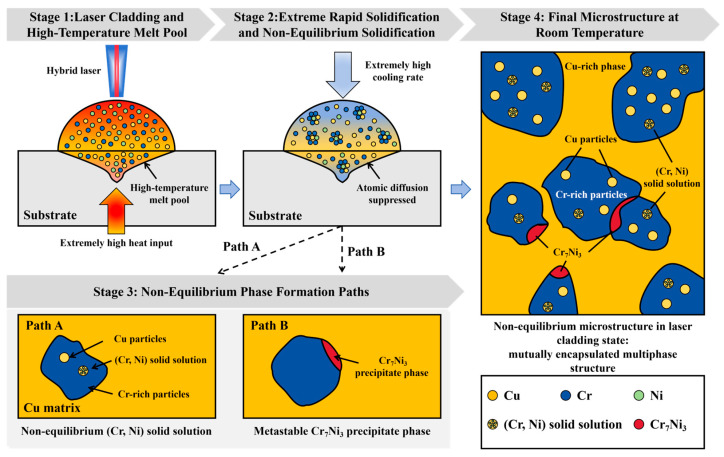
Schematic illustration of the formation and evolution of solid solution and precipitate phases during laser cladding of CuCr50Ni_0.5_.

Figure 10 shows the formation and evolution mechanisms of the (Cr, Ni) solid solution and the Cr_7_Ni_3_ phase during infrared-blue hybrid laser cladding. Under extreme non-equilibrium solidification induced by laser irradiation, the high-temperature molten pool provides the thermodynamic driving force for the diffusion and dissolution of Cr and Ni atoms within the Cu matrix. The extreme cooling rate of the molten pool substantially suppresses long-range atomic diffusion. Consequently, Cr and Ni are retained in a supersaturated solid solution within the Cu matrix, forming a metastable (Cr, Ni) phase, rather than precipitating as equilibrium intermetallics. Concurrently, trace Cu is encapsulated within the Cr phase (Path A), a phenomenon that reduces Cr particle size, consistent with the observations in Figure 6b. Meanwhile, the dissolved Cr and Ni undergo local segregation, preferentially precipitating as a metastable Cr_7_Ni_3_ phase along the boundaries of the Cr-rich phase (Path B). As seen in Figure 7c, the Cr_7_Ni_3_ precipitates preferentially nucleate and grow at the heterophase interfaces between the (Cr, Ni) solid solution and the Cu/Cr constituents, with their dimensions consistently remaining at the sub-micrometer scale. According to the Zener pinning theory, these intergranular precipitates effectively inhibit Cr grain coarsening and boundary migration. This results in a finer and more stable Cr dispersion in the CuCr50Ni_0.5_ alloy compared to the CuCr50 composite, thereby indirectly increasing its heterophase interface density. This increased interfacial density intensifies electron scattering at the phase boundaries, consequently increasing the electrical resistivity of the material [47]. Compared to the CuCr50 coating, the CuCr50Ni_0.5_ coating exhibits higher hardness but slightly lower electrical conductivity.

### 3.2. Arc Erosion Resistance of CuCr50Ni_X_ Coatings at Different Ni Contents

**Figure 11:**
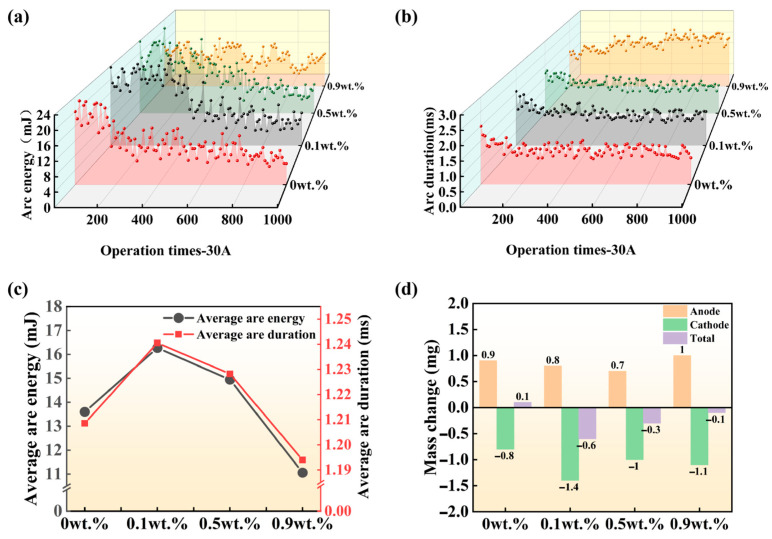
Arc characteristics and mass transfer behavior of CuCr50Ni coatings with varying Ni content under 24 V and 30 A: (**a**) arc energy profiles; (**b**) arc duration; (**c**) average arc energy and arc duration; (**d**) mass transfer evolution.

Figure 11 presents the evolution of arc energy and arc duration over successive switching operations, along with average arc parameters and post-test mass transfer behavior, for laser-clad CuCr50Ni composites with varying Ni content, tested at 24 V and 30 A. As shown in Figure 11a,b, both arc energy and arc duration exhibit a fluctuating upward trend with increasing switching cycles, with a strong correlation between their variations. Specifically, the CuCr50 sample shows arc energy stabilized within 8–16 mJ and arc duration maintained at 1.0–1.5 ms. When the Ni content rises to 0.5%, arc energy increases to 12–20 mJ and arc duration remains stable at around 2.0 ms. Notably, the CuCr50Ni_0.5_ sample exhibits the smallest fluctuation in both arc energy and arc duration, indicating superior arc stability. However, when the Ni content reaches 0.9 wt.%, the arc parameters become markedly more erratic, revealing that excess Ni compromises arc stability. Figure 11c shows the average arc energy and arc duration for CuCr50Ni coatings with different Ni contents. Both parameters exhibit a pronounced peak at 0.1 wt.% Ni. Subsequently, as the Ni content increases further to 0.9 wt.%, both values progressively decrease. This phenomenon indicates that the introduction of trace Ni (0.1 wt.%) disrupts the dispersed distribution of the Cr phase in the CuCr50 material, inducing localized microstructural inhomogeneity, which in turn exacerbates arc energy accumulation and prolongs arcing duration. As the Ni content increases, its solid solution and refinement effects within the matrix become increasingly pronounced, partially restoring microstructural uniformity and thereby mitigating arc erosion. Mass transfer analysis (Figure 11d) substantiates this mechanism: all specimens show anode mass gain and cathode mass loss, indicating pronounced directional material transfer from cathode to anode during electrical contact. The greatest cathode mass loss (−1.4 mg) occurs at a Ni content of 0.1 wt.%, coinciding with the most severe arc erosion. At a Ni content of 0.9 wt.%, cathode loss declines to −1.1 mg, anode gain increases from 0.8 mg to 1 mg, and the net mass change drops from −0.6 mg to −0.1 mg. This trend closely aligns with the evolution of arc parameters, confirming that Ni content, by modulating microstructural uniformity, directly governs the arc erosion resistance and mass transfer behavior of the clad layer. In summary, the results reveal that Ni optimization in the CuCr50Ni clad layer must reconcile uniformity and arc stability. Both excessive and insufficient Ni additions exacerbate arc erosion and mass loss. At an optimal Ni content of 0.5 wt.%, the material maintains favorable electrical conductivity while exhibiting superior arc erosion resistance.

**Figure 12:**
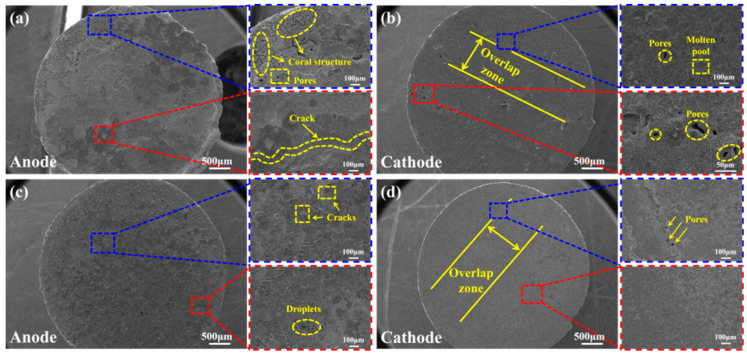
Arc erosion morphology of CuCr50 and CuCr50Ni_0.5_ coatings at 24 V and 30 A: (**a**) CuCr50 anode; (**b**) CuCr50 cathode; (**c**) CuCr50Ni_0.5_ anode; (**d**) CuCr50Ni_0.5_ cathode.

Figure 12 shows a comparative analysis of the anode and cathode arc erosion morphologies of CuCr50 and CuCr50Ni_0.5_ under electrical contact conditions of 24 V and 30 A. As shown in Figure 12a, the anode surface of the CuCr50 specimen exhibits pronounced non-uniform ablation characteristics, featuring continuous cracks, typical coral-like protrusions, and numerous pores. In contrast, the cathode surface (Figure 12b) shows discernible pores near the cladding overlap region, yet it maintains a relatively flat morphology without significant cracking. With 0.5 wt.% Ni addition (Figure 12c), the anode achieves substantially enhanced surface flatness, featuring only fine cracks and metallic spatters, free from coral-like features and macropores. Correspondingly, the cathode surface (Figure 12d) displays discernible pores and small molten pools in the overlap region, albeit with substantially reduced pore size and density. These morphological differences are attributable to the modulating effect of Ni addition on microstructural homogeneity and arc behavior. In the absence of Ni, the Cr phase particles remain relatively coarse and agglomerated, promoting preferential arc root attachment to Cr-rich regions. The resulting localized heat accumulation induces rapid melting and vaporization of the Cu matrix, thereby forming molten pools. During solidification of these molten pools, dissolved gases (e.g., O_2_ and N_2_) are expelled, resulting in surface porosity and the characteristic coral-like solidification structures [48,49]. Meanwhile, the differential cooling rates between Cu and Cr generate thermal stresses that promote crack initiation at the periphery of the molten pool [50]. The cathode, as the source of electron emission, exhibits slightly higher arc root mobility compared to the anode. The overlap zone on the cathode surface comprises unmelted Cr particles embedded in the Cu matrix (Figure 4(a_1_–d_1_)). This induces localized current crowding, leading to vaporization of the Cu matrix and the formation of pores. Owing to its lower heat accumulation relative to the anode, the cathode does not develop extensive molten pools or coral-like structures. In CuCr50Ni, however, the solid solution of Ni in the Cu matrix induces lattice distortion, refines the Cr-rich phases, and promotes their homogeneous dispersion. This microstructural evolution impedes sustained arc root attachment in the overlap region, instead prompting dynamic migration among finely dispersed Cr precipitates. Such behavior effectively precludes localized heat accumulation, thereby suppressing gas evolution and the formation of coral-like solidification structures on the anode surface. Only fine cracks and isolated metal droplets remain. By avoiding localized current concentration, this also reduces the vaporization rate of the Cu matrix in the overlap region of the cathode, ultimately resulting in the formation of discrete pores rather than interconnected defects on the cathode surface. The presence of (Cr, Ni) solid solutions and trace Cr_7_Ni_3_ precipitates within the Cr phase (Figure 7c) mitigates localized superheating under arc discharge conditions. Concurrently, these precipitates increase the density of phase boundaries, impeding molten pool convection and crack propagation. This constrains anodic erosion to a shallow and uniform morphology. Furthermore, the precipitates reinforce the mechanical integrity of the cathodic surface, mitigating material exfoliation induced by arc thermal stress. As a result, porosity remains isolated and localized, preventing widespread morphological degradation.

**Figure 13:**
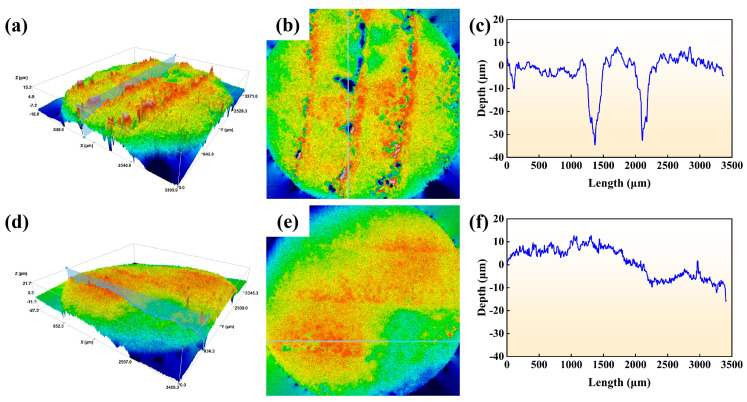
Three-dimensional topography and profile depth of laser-clad CuCr50 cathodes and CuCr50Ni_0.5_ cathodes: (**a**–**c**) CuCr50 cathode; (**d**–**f**) CuCr50Ni_0.5_ cathode.

Figure 13 presents the three-dimensional topography and corresponding depth profiles of cathodes fabricated via laser cladding of CuCr50 and CuCr50Ni_0.5_, under conditions of 24 V and 30 A. Using the pristine, uneroded surface as a reference, the chromatic map encodes depressions in blue and protrusions in red, with color gradients directly visualizing post-ablation roughness distribution. As depicted in Figure 13a–c, the CuCr50 cathode displays a markedly undulated surface morphology. The interwoven distribution of deep-blue concavities and red protrusions in the chromatic mapping reveals a distinct localization characteristic of the arc ablation process. Profile analysis corroborates a severe erosion pit, approximately 37.7 μm in depth, near the cladding overlap zone (Figure 13c), signifying substantial localized material loss. In contrast, the CuCr50Ni_0.5_ cathode exhibits substantially reduced surface roughness (Figure 13d–f). The chromatic distribution is notably homogenized, devoid of extreme concavities or convexities. The maximum pit depth is only 9.6 μm (Figure 13f), and the eroded area exhibits lateral expansion, manifesting a shallow and broad uniform erosion morphology. This finding demonstrates that Ni addition markedly enhances arc distribution uniformity across the cathode surface, mitigating localized ablation. These three-dimensional topography observations align with the SEM analysis in Figure 12, collectively affirming the superior arc erosion resistance and more stable discharge behavior of the CuCr50Ni alloy.

### 3.3. Arc Erosion Resistance Mechanisms of the CuCr50 and CuCr50Ni_0.5_ Coatings

Based on the microstructural analysis presented in Figure 4, the addition of 0.5 wt.% Ni refined the average grain size of the Cr phase to 2.16 ± 0.15 μm. TEM observation (Figure 7) further revealed a small number of dispersed Cr_7_Ni_3_ nanoprecipitates (approximately 5–15 nm) along the boundaries of the refined Cr phase. These precipitates effectively inhibit Cr phase coarsening during solidification and subsequent arc exposure by pinning grain boundaries. Experimental results reveal that the ablation resistance of laser-clad CuCr50 and CuCr50Ni_0.5_ materials is closely correlated with the synergistic effects involving the distribution of Cr phases, (Cr, Ni) solid solutions, and Cr_7_Ni_3_ precipitates. This study systematically elucidates the evolution mechanisms of arc ablation behavior for both materials under 24 V and 30 A conditions via a schematic mechanistic diagram.

**Figure 14:**
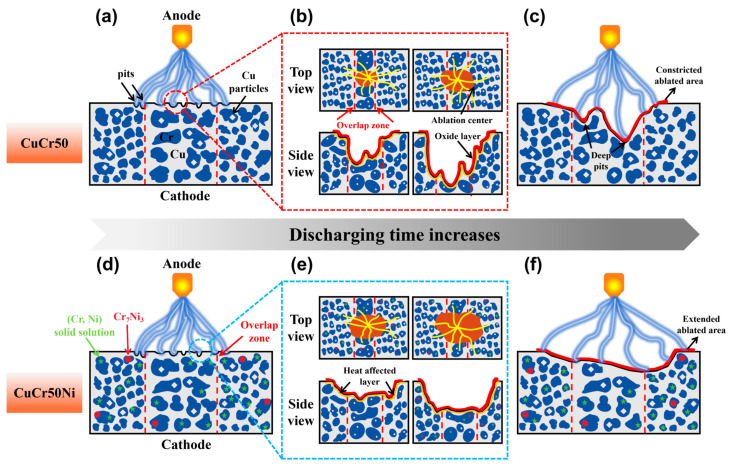
Schematic diagram of the arc erosion mechanism for CuCr50 and CuCr50Ni_0.5_ coatings: (**a**–**c**) CuCr50; (**d**–**f**) CuCr50Ni_0.5_.

As shown in Figure 14, when an arc is initially discharged on the material surface, slight ablation traces appear on the surfaces of both materials. With extended discharge duration, ablation pits on both materials are predominantly located near the overlap zone. Notably, the ablation pits on CuCr50 cathodes display a narrow and deep morphology, whereas those on CuCr50Ni_0.5_ cathodes exhibit a uniform wide and shallow profile. Studies indicate that for CuCr alloys, cathode breakdown tends to occur preferentially in the Cr phase, which exhibits a lower breakdown strength and acts as the preferential discharge phase [51]. As shown in Figure 14a, for the CuCr50 clad layer, Cr particles in the overlap zone are coarse and clustered, while those elsewhere remain densely packed and well-ordered. Coupled with the lower work function of Cr (~4.5 eV) relative to Cu (~4.65 eV), this renders the Cr phase a favorable site for initial arc breakdown [52,53]. During ablation, the arc root preferentially attaches to the coarse Cr-rich regions within the overlap zone, inducing localized heat concentration. The adjacent Cu matrix, with its lower melting point (1085 °C), undergoes rapid melting and vaporization, further exacerbating thermal accumulation. Sustained discharge subjects the exposed Cr phase to high-temperature ablation, generating localized cavities that progressively evolve into deep, vertically oriented ablation pits (Figure 14b). As the discharge process proceeds, the ablation zone is restricted by the inhomogeneous distribution of the Cr phase. The arc root is confined to the vicinity of coarse Cr phases with a very limited moving range, causing the erosion to extend inward along the breakdown path. This culminates in a characteristic erosion morphology featuring localized, deep, and constricted ablation pits (Figure 14c).

Figure 14d shows that for CuCr50Ni with 0.5 wt.% Ni addition, solid solution refinement by Ni effectively reduces Cr phase size and enhances its dispersion, while inducing nanoscale Cr_7_Ni_3_ precipitation at Cr phase boundaries. This microstructural feature enables the arc root to move dynamically among numerous fine Cr phases, significantly expanding its movement range and thereby preventing localized heat accumulation. Moreover, the synergistic interaction between the (Cr, Ni) solid solution and the Cr_7_Ni_3_ precipitate improves the homogeneity of local thermal conductivity. This leads to a more progressive melting and vaporization of the Cu matrix, markedly diminishing gas evolution and thermal stress concentration resulting from intense thermal shock. According to the Stokes law [54], the motion behavior of molten metal droplets in the pool can be described by the following equation:

(3) $$F_{d}=3\pi \mu d\upsilon_{\infty }$$

Here, *F_d_* is the frictional resistance, *μ* is the viscosity of the molten pool, *d* is the particle diameter, and *υ_∞_* is the flow velocity. As indicated by this equation, an increase in molten pool viscosity significantly reduces the droplet splashing rate. Ni atoms dissolve into the cladding layer, thereby increasing the molten pool viscosity and effectively suppressing both the splashing rate and violent melt flow. Furthermore, the presence of (Cr, Ni) solid solutions and Cr_7_Ni_3_ precipitates enhances the interfacial stability of the Cr phase, exerting a pinning effect on molten pool flow. During discharge, the arc extends outward along dispersed Cr phases beyond the overlap zone, uniformly broadening the erosion area with no localized deep erosion (Figure 14e). Owing to their high melting point and exceptional thermal stability, the Cr_7_Ni_3_ precipitates effectively impede arc penetration into deeper material regions, substantially reducing the ablated layer thickness. This culminates in a uniformly shallow ablation morphology, as evidenced in Figure 14f. In summary, the CuCr50Ni_0.5_ clad layer, through the combined mechanisms of Ni solid solution refinement and interfacial strengthening via Cr_7_Ni_3_ precipitation, achieves markedly enhanced microstructural uniformity. This governs the arc attachment and propagation behavior, culminating in a systematic improvement in the material’s resistance to arc ablation.

Through systematic electrical contact performance experiments, this study demonstrates that the addition of an appropriate amount of Ni into CuCr50 alloy significantly enhances the arc erosion resistance of its cathode material, thereby effectively extending the service life of electrical contact components. However, despite the progress made in composition optimization, the current laser cladding process still exhibits certain limitations. The main issues involve microstructural inhomogeneities such as Cr phase coarsening in the cladding overlap zone and inadequate precision in secondary phase distribution, which collectively impede further enhancement of overall material performance. To overcome these limitations, subsequent research should focus on optimizing laser processing parameters, particularly key fabrication parameters such as overlap ratio. It should also focus on achieving a homogeneous composite coating on Cu substrates with refined grains and controllable reinforcement distribution, without compromising the inherent high electrical conductivity. Consequently, future efforts will focus on developing advanced, precision surface engineering strategies to synergistically optimize arc ablation resistance and electrical conductivity in cathode materials. This will establish a novel technological and theoretical framework for next-generation electrical contacts with superior longevity and reliability.

## 4. Conclusions

In this study, CuCr50Ni_X_ (X = 0, 0.1, 0.5, and 0.9 wt.%) coatings were prepared on pure copper substrates using infrared-blue hybrid laser cladding technology. The regulatory mechanism of Ni on the microstructure, mechanical properties, electrical conductivity, and arc erosion resistance of the coatings was systematically investigated. Comparative analysis of coatings with different Ni contents revealed the intrinsic correlations between microstructural evolution and macroscopic properties, and further clarified the failure behavior and strengthening mechanisms under arc discharge. The main conclusions can be summarized as follows:

1. The addition of Ni refines the Cr phase microstructure in the coating and promotes the formation of a (Cr, Ni) supersaturated solid solution, effectively suppressing Cr phase growth and aggregation. The average Cr grain size is refined to 2.16 ± 0.15 μm at a Ni content of 0.5 wt.%, representing a reduction of approximately 3.5% compared to the Ni-free coating. Under extreme non-equilibrium solidification during laser cladding, Ni atoms dissolve into the Cu matrix and Cr phase, with nanoscale Cr_7_Ni_3_ precipitates forming at the Cr/Cu phase boundaries, thereby enhancing interfacial bonding and improving coating structural stability.
2. At a Ni content of 0.5 wt.%, the coating achieves an optimal synergy between hardness and electrical conductivity. The combined effects of solid solution and grain refinement strengthening elevate the average microhardness of the coating to 174.2 HV_0.5_, representing a 149% enhancement relative to the substrate. Although Ni solutes induce lattice distortion and intensify electron scattering, the refinement and homogeneous dispersion of the Cr phase mitigate interfacial electron scattering at phase boundaries, thereby preserving the electrical conductivity of 29.8% IACS.
3. Electrical contact tests demonstrate that the coating with 0.5 wt.% Ni addition exhibits superior arc erosion resistance. Compared with the CuCr50 coating, the cathode mass loss is reduced from −1.4 mg to −1.1 mg (21% reduction in mass loss), accompanied by diminished fluctuations in both arc energy and arc duration. The surface erosion morphology transitions from localized narrow and deep ablation pits (~37.7 μm) to uniform shallow and broad ablation pits (~9.6 μm). Furthermore, the dissolution of Ni increases the viscosity of the molten pool, effectively suppressing melt splashing and violent flow. Meanwhile, the (Cr, Ni) solid solution and Cr_7_Ni_3_ precipitates enhance high-temperature phase stability and thermal conductivity uniformity, synergistically suppressing severe melting and vaporization of the Cu matrix in the cladding overlap zone, thereby preventing the formation of pores, cracks, and coral-like remelted structures.

## Figures and Tables

**Table 1:** Typical processing parameters used in the experiments.

Blue Laser Power (W)	Infrared Laser Power (W)	Scanning Speed (mm/s)	Powder Feeding Rate (g/min)	Overlap Ratio (%)
1800	1200	10	12	50

**Table 2:** Lattice constant and relative distortion of CuCr50 coatings with different Ni contents.

Materials	Cu-Rich Phase Diffraction Peaks	d (nm)	a (nm)	Da (%)
CuCr50	(111)	0.22422	0.38836	0
CuCr50Ni_0.1_	(111)	0.22447	0.38879	0.11072
CuCr50Ni_0.5_	(111)	0.22449	0.38883	0.12102
CuCr50Ni_0.9_	(111)	0.22454	0.38891	0.14162

## Data Availability

The original contributions presented in this study are included in the article. Further inquiries can be directed to the corresponding authors.
